# Characterization of Ebinur Lake Virus and Its Human Seroprevalence at the China–Kazakhstan Border

**DOI:** 10.3389/fmicb.2019.03111

**Published:** 2020-01-30

**Authors:** Han Xia, Ran Liu, Lu Zhao, Xiang Sun, Zhong Zheng, Evans Atoni, Xiaomin Hu, Bo Zhang, Guilin Zhang, Zhiming Yuan

**Affiliations:** ^1^Key Laboratory of Special Pathogens and Biosafety, Wuhan Institute of Virology, Chinese Academy of Sciences, Wuhan, China; ^2^Illumina (China), Beijing, China; ^3^University of Chinese Academy of Sciences, Beijing, China; ^4^Center for Disease Control and Prevention of Xinjiang Uygur Autonomous Region, Urumqi, China

**Keywords:** Xinjiang, Ebinur Lake virus, *Culex modestus*, mosquito, *Orthobunyavirus*

## Abstract

In recent years, rapidly increasing trade and travel across the China–Kazakhstan border has increased the potential risk of the introduction and exportation of vectors and their related diseases. The Ebinur Lake Nature Reserve is located in Xinjiang Uygur Autonomous Region, near the China–Kazakhstan border, with a suitable ecosystem for mosquito breeding. In our previous work, a novel *Orthobunyavirus* species named Ebinur Lake virus (EBIV) was isolated in the reserve. To gain insights into the potential risk of EBIV in this region, we conducted a study that aimed to clearly outline EBIV’s biological characteristics and its human seroprevalence in this region. Phylogenetically, the analysis of all three segments of EBIV demonstrated that it belongs to the genus *Orthobunyavirus*, which is clustered in the Bunyamwera serogroup. EBIV replicated efficiently and caused cytopathic effects (CPEs) in vertebrate cells. The survival rates of the EBIV-challenged mice were 0 and 20% when inoculated with viral concentrations ≥10^4^ or 10^2^ plaque-forming units, respectively. For EBIV-infected mice, internal bleeding and pathological changes were observed. In addition, the overall immunoglobulin M (IgM) antibody [1:4 by immunofluorescence assay (IFA)], immunoglobulin G (IgG) antibody (1:10 by IFA), and neutralizing antibody [90% plaque reduction neutralization test (PRNT)] prevalence was 8.05, 12.3, and 0.95%, respectively, in the studied residents. In summary, EBIV is a new member of the Bunyamwera serogroup and is able to competently infect cells derived from mosquitoes, rodents, monkeys, or humans. Furthermore, EBIV caused severe disease and even death in challenged Kunming mice, and the antibodies against EBIV have been detected in local residents, indicating that the virus is a potential animal or human pathogen.

## Introduction

Mosquito-borne viruses are naturally maintained in sylvatic and urban cycles of mosquitoes, as vectors, and susceptible vertebrate hosts, as reservoirs or dead-end hosts (Hollidge et al., 2010). However, studies that focus on neglected mosquito-borne viruses are still inadequate since much attention and priority has been given to classical arboviruses that have previously caused outbreaks.

Ebinur Lake, the largest saltwater lake in western Xinjiang Uygur Autonomous Region, China, is located within the Ebinur Lake Wetland National Nature Reserve (ELWNNR) (44°50′ N, 82°50′ E) near the China–Kazakhstan border. It occupies an area of ∼650 km^2^, with a 2–4 m water depth, and the average altitude is over 189 m. Inside the reserve, its ecology is almost undisturbed and has a complex biome structure and diverse habitats, such as small water bodies, swamps, and meadows. At present, there are hundreds of bird species, and numerous wild animals and plants have been found in this reserve, which has become an important component of China’s biodiversity and a natural gene pool (Ma et al., 2010). In addition, Ebinur Lake is used as the main staging habitat during the migration of birds in Central Asia, where at least 200,000 water birds reside annually in the autumn (Ma et al., 2010). Hence, it provides a conducive habitat and suitable ecosystem for mosquito breeding and a virus–vector–host cycle. In recent years, the China–Kazakhstan border has become a crucial region that connects China with countries in Central Asia and Europe. As a result, trade and travel across this region have rapidly increased, thus increasing the potential risk of the introduction and exportation of vectors and related diseases (The Lancet Global Health, 2017; Chen et al., 2019). However, at present, little is known regarding arboviruses associated with mosquitoes in the Ebinur Lake Wetland National Nature Reserve.

The family *Peribunyaviridae* was created to include the established bunyaviral genera *Orthobunyavirus*, *Herbevirus*, and *Pacuvirus* and the newly defined genus, *Shangavirus* (Abudurexiti et al., 2019). Currently, *Orthobunyavirus* is the largest genus within the *Peribunyaviridae* family and is distributed worldwide; it has a trisegmented negative-sense RNA genome comprising small (S), medium (M), and large (L) segments (Elliott, 2014). Reassortment and recombination are two important mechanisms in segmented RNA viruses, since these processes expand their viral genetic diversity and may significantly alter the virulence or other related biological properties (Gentsch et al., 1979; Lukashev, 2005; Briese et al., 2006, 2013; Shi et al., 2017; Nunes et al., 2019). Most orthobunyaviruses can infect vertebrates and exist in a zoonotic infection cycle, in which the transmission between humans and animals occurs via an arthropod intermediate. Human infections caused by viruses from the genus *Orthobunyavirus* can result in acute, mild febrile illnesses (Bunyamwera virus) to encephalitis (California encephalitis virus, La Crosse virus, or Tahyna virus) and hemorrhagic fevers (Ngari virus). In the past decade, novel orthobunyaviruses associated with human and/or livestock diseases (Iquitos virus, Itaya virus, Ntwetwe virus, and Schmallenberg virus) have been identified. Furthermore, known orthobunyaviruses have re-emerged or expanded to new geographical territories, a situation that has presented a serious economic and public health threat (Aguilar et al., 2011; Wernike and Beer, 2017; Edridge et al., 2018).

From our previously conducted mosquito-borne virus surveillance study in the Ebinur Lake region in 2014, a novel orthobunyavirus, Ebinur Lake virus (EBIV), which was previously named Abbey Lake virus, was isolated from *Culex modestus* mosquito pools, and its whole genome sequences were reported (Liu et al., 2014b, c). However, its detailed characterization and impact on animal or human health have not been investigated. Therefore, in the present study, we aimed to (1) extensively conduct EBIV’s molecular analysis and outline its phylogenetic classification, (2) determine its *in vitro* infectivity range on various cell lines and *in vivo* infectivity pattern through the use of a mouse model, and (3) conduct an EBIV human seroprevalence study in the Ebinur Lake region. Certainly, our findings provide and subsequently expand the much-needed knowledge on EBIV, and this information can be utilized in the prevention and control of this neglected potential zoonotic virus.

## Materials and Methods

### Ethics Statement

Animal studies were approved by the Animal Care and Use Committee of the Center for Disease Control and Prevention of Xinjiang Military Command Region (Approval No. 2014001). Human serum collection was approved by the Ethical Committee of the Center for Disease Control and Prevention of Xinjiang Military Command Region (Approval No. 2014006). All adult subjects provided informed consent, which was given in writing, and a parent or guardian of any child participant provided informed consent on the child’s behalf.

### Cell Lines, Virus Stocks, and Animals

*Aedes albopictus* C6/36 cell lines were grown in Roswell Park Memorial Institute 1640 medium with 1% penicillin/streptomycin and 10% fetal bovine serum at 28°C. BHK-21, Vero, and SW13 cells were grown in Dulbecco’s modified Eagle’s medium with 1% penicillin/streptomycin and 5–10% fetal bovine serum (Gibco, United States) at 37°C in 5% CO_2_.

The EBIV isolate Cu-XJ20 was originally isolated from *C. modestus* mosquitoes in 2013 in Xinjiang, China, by inoculation in suckling mice and passage three times in BHK-21 cells as a seed stock. Then, the working stock was generated, and the titer was 1.2 × 10^7^ plaque-forming units/ml (PFU/ml).

Kunming mice, progeny of Swiss mice that are regarded as a viral infection animal model (Yu et al., 2017), were used in this study. Mice aged 6–8 weeks (20–25 g) were used since they have potent immune responses at this point. The mice were acquired from the Animal Center of Xinjiang Medical University and maintained in an ABSL-2 facility with controlled temperature (22°C), humidity, and a 12-h light/dark cycle.

### Seroprevalence Study Sites and the Collection of Serum Samples

**FIGURE 1:**
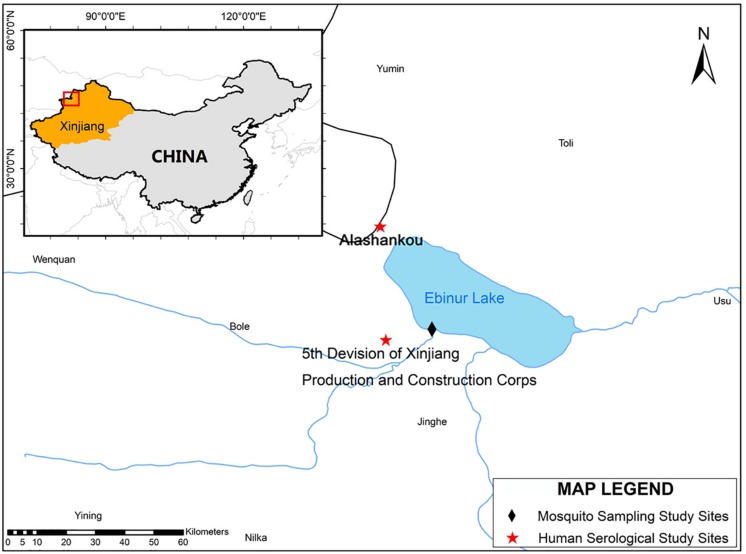
The mosquito collection and serological study sites map. The map and the associated shapefiles were constructed and obtained from the free and open source Quantum GIS software (https://qgis.org/en/site/) and DIVA-GIS platform (https://www.diva-gis.org/gdata).

Alashankou and the Fifth Division of Xinjiang Production and Construction Corps were selected as human seroprevalence study sites. These two study sites are approximately 39 and 21 km, respectively, from the site where EBIV was originally isolated from mosquitoes (Figure 1).

Between 2014 and 2015, a total of 211 human serum samples were collected from residents who visited the local outpatient clinic during the high breeding season of mosquitoes. Study participants were divided into two groups: one group comprised individuals with no clinical symptoms visiting regular physical examinations, while the other group comprised individuals with fever (>37.5°C) of unknown origin. The samples were collected within 1–3 days of the onset of clinical symptoms. Sociodemographic characteristics of the study population, including gender, age, location, and occupation, were collected from every study participant.

### Bioinformatics Analyses

The complete coding sequences (CDSs) of all 3 segments of 55 strains (except the Germiston virus, which lacks L segment information) belonging to the serogroups of Bunyamwera, California, Group C, Guma, Mapputta, Patois, and Simbu in the *Orthobunyavirus* genus together with the representative members of Herbevirus (Herbert virus) were used in the analysis (from GenBank as of 28 December 2018). Background information of all the utilized strains is listed in Supplementary Table 1.

The nucleotide and amino acid sequence identities of the CDSs of EBIV and other orthobunyaviruses were analyzed using SDT1.2^1^. Alignment of EBIV with the representative members of the Peribunyaviridae family was conducted by the ClustalW function in MEGAv7.0.21^2^. The GTR + I + G substitution model was selected as the best-fit nucleotide substitution model by jModelTest 2^3^. Phylogenetic analyses among nucleotide sequences were determined by the maximum likelihood method using the general time-reversible model with 1,000 bootstrap replicates in MEGA.

The phylogenetic trees and the percentage identities were evaluated to identify possible reassortment. The recombination Detection Program-4 (RDP4 v. 4.74)^4^ was used to rule out intrasegment recombination.

### Electron Microscopy

Viral particle purification was performed as previously described (Wang et al., 2017; Xia et al., 2018a). Electron microscopy imaging was performed using the negative contrast method. Pure virus particles were added to a Formvar carbon-coated copper grid for 10 min and negatively stained for 2–3 min with 2% phosphotungstic acid with the pH adjusted to 6.8 with 1 M KOH. Then, the samples were examined with a Hitachi U8010 electron microscope (Japan).

### Plaque Assay

Prepared aliquots of 10-fold serial dilutions of EBIV in Dulbecco’s modified Eagle’s medium were inoculated onto the BHK-21 cell monolayers in 24-well plates for 1 h. The cells were then covered with an overlay medium including 1.5% methylcellulose and incubated at 37°C for 3–4 days to allow plaque development. Afterward, the infected cells were stained with 2% crystal violet in 30% methanol for 5 min at room temperature. The plaques were manually counted and measured.

### Growth Curves of EBIV in Cultured Cells

C6/36, BHK-21, Vero, and SW13 cells grown in T75 flask plates were infected with EBIV at the multiplicity of infection (MOI) of 0.0001, 0.01, and 1. After inoculation, 2 ml supernatant was collected daily (day 1–7) from the T75 flask and then replenished with 2 ml fresh medium. Viral titers of the collected samples were detected via plaque assay with BHK-21 cells. This experiment was repeated three times.

### Mice Challenge and Pathogenesis Experiments

For the survival experiment, three groups of Kunming mice (five per group) were inoculated intraperitoneally (i.p.) with EBIV at 10^6^, 10^4^, and 10^2^ PFU (200 μl per mouse), and one group (three per group) was mock infected. The experiment was repeated three times. The infected mice were observed daily until death, and during this observation period, their clinical symptoms and weight changes were recorded. For pathogenesis experiments, six mice were inoculated with EBIV at 10^3^ PFU through the i.p. route. At the point of death, the mice were anesthetized, and the blood, brain, liver, kidney, spleen, and intestine were collected for virus titration and pathological examination. To determine viral titers in tissues, 1 mg of tissue was removed during necropsy, homogenized in 200 μl of phosphate-buffered saline (PBS), and then used in RNA extraction. Tissues used for pathological examination were immediately fixed with 10% paraformaldehyde for 16–24 h and embedded in paraffin, where they were processed and sectioned before staining with hematoxylin and eosin (H&E). Thereafter, the obtained sections of EBIV-infected and mock-infected samples were viewed by light microscopy to analyze the histopathological changes. This was performed at the Department of Pathology, Center for Disease Control and Prevention in Xinjiang.

### Immunofluorescence Assay

Vero cells infected with EBIV were cultured for 4 days and then harvested by trypsinization before being washed with PBS. The cells were spotted onto 14-well HT-coated glass slides and fixed with acetone at room temperature for 5 min, and the slides were then stored at −80°C until use. Serum samples were diluted with PBS to 1:4 and 1:10. The diluted samples were placed on EBIV slides and incubated under humidified conditions at 37°C for 1 h. Next, the slides were washed three times with 0.01 M PBS (pH 7.4). Fluorescein isothiocyanate-conjugated goat antihuman immunoglobulin G (IgG) or immunoglobulin M (IgM) antibody was then added (200 μl/well), and the slides were incubated at 37°C for 30 min and rinsed with PBS to remove excess secondary antibody. The slides were visualized using a fluorescence microscope (Olympus, Japan). Normal human serum purchased from ImumunoReagents Inc. was also tested as the negative control under the same conditions in each immunofluorescence assay (IFA) test for verification.

### Neutralizing Antibody Test

Immunofluorescence assay-positive samples were each tested using a plaque reduction neutralization test (PRNT) assay for EBIV. Briefly, sera were heat inactivated at 56°C for 30 min and diluted to 1:4, followed by twofold serial dilutions. Sera were then mixed with 100 PFU of virus and incubated at 37°C for 1 h. The virus–serum dilution mixtures were then inoculated into BHK-21 cell monolayers in 24-well plates for 1 h before adding an overlay. After 3 days of incubation at 37°C, the plates were stained, and plaques were counted. All positive samples were further titrated to determine endpoint titers. The PRNT titer was calculated based on a 90% reduction in plaque count (PRNT90).

### RNA Extraction and Quantitative Real-Time RT-PCR

To determine the viral RNA in mouse blood or tissue, 20 μl serum (separated from 100 μl blood) with 130 μl PBS or 150 μl tissue homogenization supernatant was mixed with 450 μl TRI reagent. RNA was extracted using Direct-zol RNA MiniPrep (Zymo Research, United States) following the manufacturer’s instructions. The S segment was cloned into the plasmid vector pBlunt with a T7 promoter (Wuhan Tianyi Huiyuan Bioscience & Technology Inc., China) and then linearized by restriction enzyme digestion as a template for *in vitro* RNA transcription using the HiScribe^TM^ T7 Quick High-Yield RNA Synthesis Kit (NEB, United States) according to the manufacturer’s instructions. The transcripts were purified using the RNeasy Plus Mini Kit (Qiagen, Germany) and resuspended in 40 μl of RNase-free water. The concentrations of *in vitro* transcribed RNAs were determined using a NanoDrop ND-2000 (Invitrogen, United States) and used as the RNA standard.

Ebinur Lake virus-specific quantitative real-time reverse transcription PCR (RT-PCR) was performed using the primers and probes targeting the S segment as follows: probe (5′-FAM-TTTTGGGTCCATCTCTTTCCTCTGC-TAMRA-3′) and primers (forward: 5′-GGTACCTCTGGCGCATTGTCTTTTC-3′ and reverse: 5′-GAAAAATGGCATCACCTGGGAAAGT-3′). The reaction was performed on a LightCycler v2.5 platform (Roche, Switzerland) using a PrimeScript RT-PCR Kit (Takara, China) in accordance with the manufacturer’s instructions.

### Statistical Analyses

All obtained statistical data were analyzed with R (version 3.5.1)^5^. The average survival time, percentage mortality, and Kaplan–Meier survival curves were obtained using the “survival” and “KMsurv” packages. One-way ANOVA was used to determine significant differences among groups of highest viral titers in different cell lines and viral RNA copies in blood and tissue of EBIV-challenged mice.

## Results

### Molecular Phylogeny, Reassortment, and Recombination Analysis

The phylogenetic relationships among EBIV, other members in the selected serogroups of *Orthobunyavirus* (Bunyamwera, California, Group C, Guama, Mapputta, Patois, and Simbu serogroup), and Herbert virus (the outgroup) were analyzed based on all three segments, except the Germiston virus, which only has complete CDSs for the S and M segments in GenBank.

**FIGURE 2:**
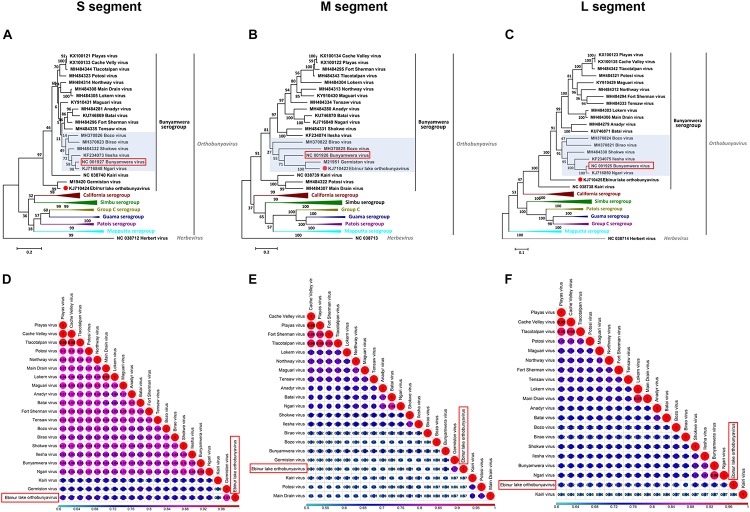
Maximum likelihood phylogenetic trees **(A–C)** and pairwise distance **(D–F)** based on alignments of nucleotide sequences of orthobunyaviruses. The scale bar indicates the evolutionary distance in the number of substitutions per nucleotide substitution/site, and the principal bootstrap support levels are indicated. Branches are color coded according to group. The Herbert virus was used as an outgroup in the ML trees.

The phylogenetic results of all three segments demonstrate that EBIV belongs to the genus *Orthobunyavirus* and clusters with the members of the Bunyamwera serogroup (Figures 2A–C). The nucleotide identity analysis matrix, based on the selected viruses, indicates that the S segment has the highest identity, whereas the M segment has the lowest identity within the Bunyamwera serogroup. The S, M, and L segments of EBIV share the highest sequence similarity with those of Germiston virus (90.7%), Germiston virus (77.3%), and Bunyamwera virus (72.7%), respectively (Figures 2D–F). The amino acid similarity analysis indicated that the amino acid sequences of the NP, GP, and L proteins of EBIV have the highest similarity with those of Germiston virus (95.7%), Germiston virus (89.0%), and Bunyamwera virus (80.3%), respectively.

The overall topology of the obtained phylogenetic trees with the S and L segment sequences was similar, as EBIV clustered with the Germiston virus or alone formed an independent clade separate from the Bunyamwera virus-clade (highlighted in a light purple color in Figures 2A,C) members. However, for the M segment, EBIV clustered with Germiston virus, which is located in the Bunyamwera virus clade (highlighted in a light purple color in Figure 2B), indicating that EBIV is a potential reassortant virus.

In addition, analysis of the three segments of EBIV and those of other members of the Bunyamwera serogroup revealed a recombination event involving EBIV, Birao virus, and Northway virus in the M segment (Supplementary Figure 1). No recombination was detected in the S or L segment. The recombination event was predicted using different algorithms (RDP, Bootscan, MaxChi, Chimaera) with a significance level set at *P* ≤ 0.05 (Supplementary Table 2).

### Viral Morphology and Efficient Growth of EBIV in Mosquito and Mammalian Cell Lines

**FIGURE 3:**
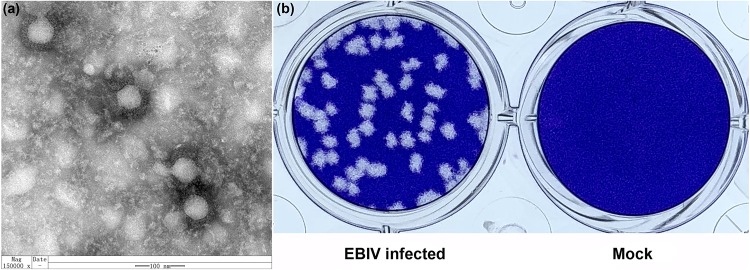
The morphology and viral plaque in cells. **(a)** Negative-stained ultracentrifuged virions of EBIV. **(b)** Crystal violet-stained BHK-21 cell monolayers showing plaques generated by EBIV at 72 h postinfection.

Mature spherical, enveloped virions of approximately 90–100 nm in diameter were detected in virus pellets generated by ultracentrifugation of EBIV-infected BHK-21 cell culture supernatants (Figure 3a), which was structurally similar to other members of the *Orthobunyavirus* genus. EBIV produced clear, ragged plaques (2–3 mm diameter) that were visible on the third day after inoculation in BHK-21 cell monolayers (Figure 3b).

**FIGURE 4:**
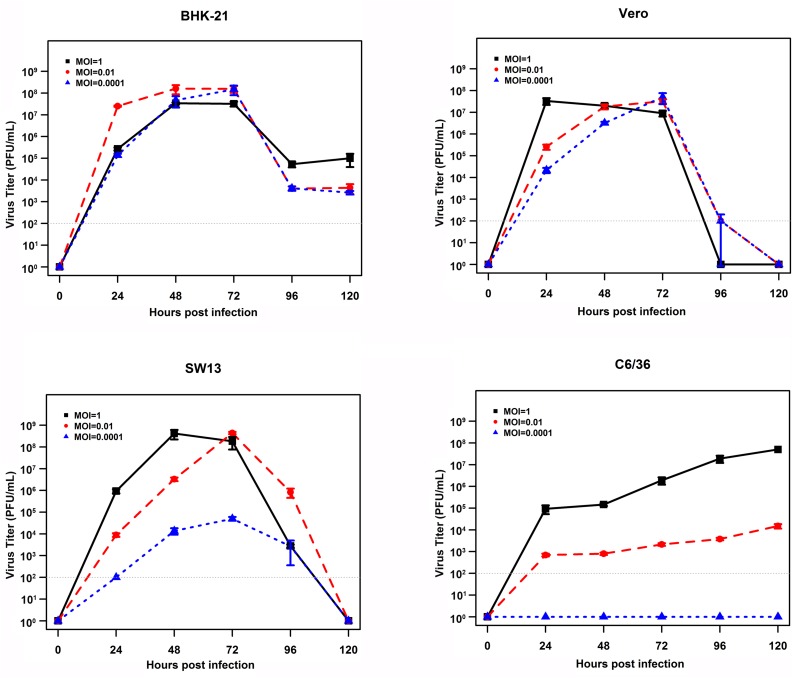
Growth curves of EBIV (MOI = 1, 0.01, and 0.0001) in cells derived from mammals and mosquitoes. A significant difference in the highest virus titers was only observed between C6/36 and BHK cells (C6/36 vs. BHK with *P* < 0.01, SW13 vs. BHK with *P* = 0.02, Vero vs. BHK with *P* = 0.03, SW13 vs. Vero with *P* = 0.99, C6/36 vs. SW13 with *P* = 0.66, and C6/36 vs. Vero with *P* = 0.55).

To gain insight into the putative host tropism, the growth of EBIV was investigated using three different mammalian cell lines derived from hamster, monkey, or human (BHK, Vero, and SW13). Prominent cytopathic effects (CPEs) were observed in all cells, which were characterized by cell shrinking, shedding, and detachment from the growth surface from days 2 to 3 (Supplementary Figure 2). The virus replicated efficiently in these cells, and the growth kinetics demonstrated that virus titers reached a peak at 10^8^ PFU/ml in BHK (at 48 h when MOI = 0.01, at 72 h when MOI = 0.0001) or in SW13 (at 48 h when MOI = 1, at 72 h when MOI = 0.01) and then drastically decreased. In Vero cells, the highest virus titers (10^7^ PFU/ml) were observed at 24 h with MOI = 1 or at 72 h with MOI = 0.01 and 0.0001. In contrast, EBIV replicated much slower in mosquito C6/36 cells, which reached 10^7^ PFU/ml at 120 h with MOI = 1, and cells exhibited very slight changes. With MOI = 0.0001, no virus could be detected between days 1 and 5 postinoculation. The significant difference (*P* < 0.01) for the highest virus titers was only observed between C6/36 and BHK cells (Figure 4).

### Mice Are Highly Susceptible to EBIV Infection

**FIGURE 5:**
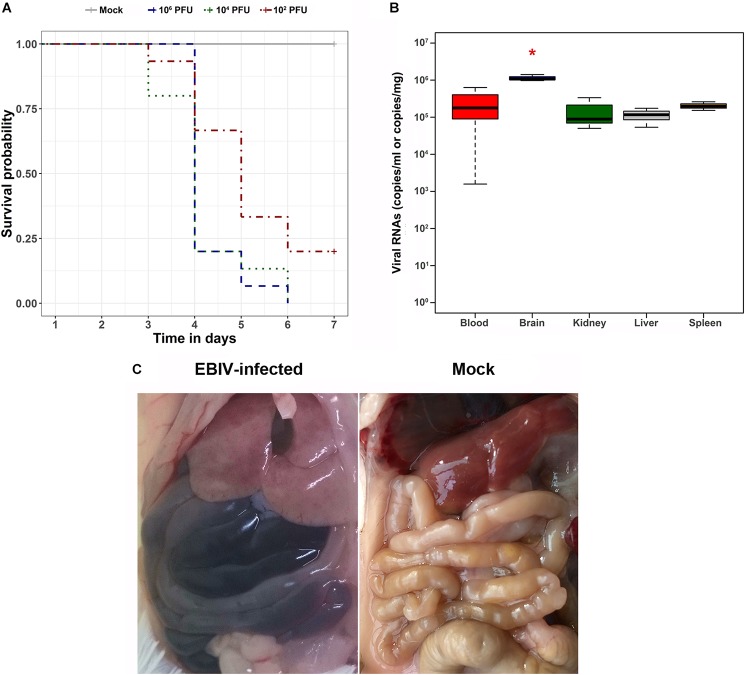
Survival analysis, viral RNA detection in tissues, and gross pathology findings of EBIV-challenged mice. **(A)** Survival curve of mice challenged with serial dilutions of EBIV. Adult mice (*n* = 5 per group, replicated three times) were challenged intraperitoneally with 10^6^ PFU (dark blue), 10^4^ PFU (dark green), and 10^2^ PFU (dark red) of EBIV. **(B)** The viral RNA copies in blood, brain, kidney, liver, and spleen tissues were detected by quantitative RT-PCR, and the brain tissue presented a significant difference (*P < 0.05) compared to the other tested tissues. **(C)** *In situ* picture of organs in EBIV-infected animals with hemorrhaging and mock-infected animals.

All mice inoculated with 10^6^ and 10^4^ PFU of EBIV per animal died within 7 days. Twenty percent of the mice in the 10^2^ PFU EBIV-inoculated group survived during the observation period (Figure 5A). The median survival time for the challenged mice was 4, 4, and 5 days in the groups treated with 10^6^, 10^4^, and 10^2^ PFU, respectively. The infected mice exhibited clinical signs of illness manifested as decreased appetite, weight loss, ruffled fur, and general weakness. The viral RNAs in the blood and tissues of the infected mice were measured using quantitative RT-PCR. The virus could replicate in the blood and all other analyzed tissues. In addition, the brain tissue had the highest level of viral RNAs, with ∼10^6^ copies/mg, which presented a significant difference (*P* < 0.001) when compared to the other tested tissues, such as liver, spleen, and kidney (Figure 5B).

During necropsy, ascites was observed in the abdominal cavities of EBIV-challenged mice. In addition, the liver appeared discolored, and the intestines harbored dark contents, indicating internal bleeding (Figure 5C), whereas the organs of the mock-infected mice appeared normal.

**FIGURE 6:**
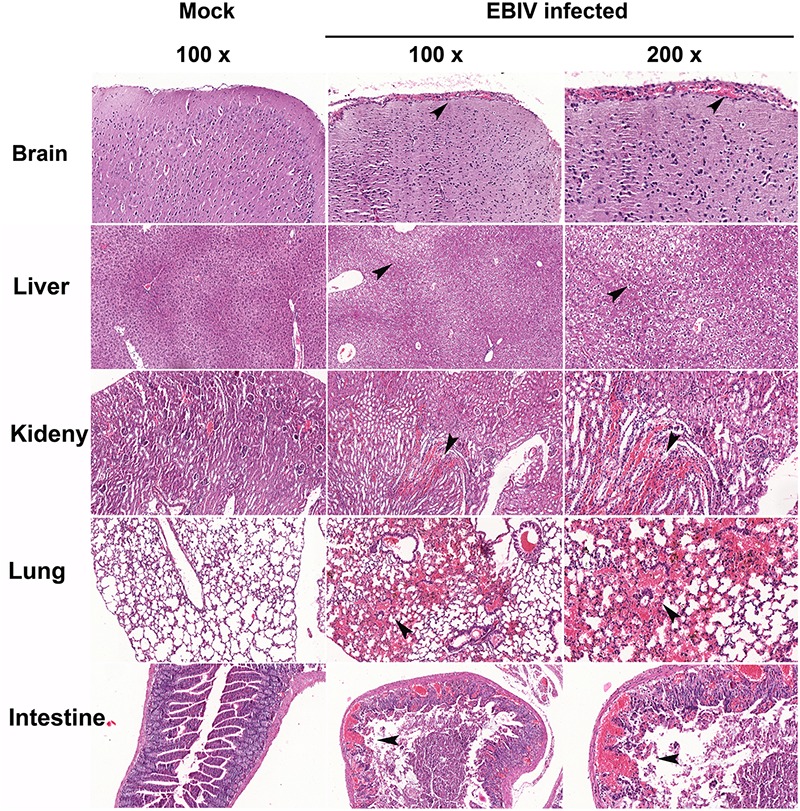
Histopathological features in the organs of EBIV-infected mice. Prominent histopathological changes in the brain, liver, kidney, lung, and small intestine, and vasodilatation and congestion of the blood vessel around meninges have been found. Hematoxylin-and-eosin-stained sections are presented. Original magnification was 100× in tissues of mock-infected, 100× and 200× in tissues infected with EBIV. The black arrows indicate histopathological changes.

To investigate the histopathological features of the EBIV-infected mice, the organs were harvested from infected mice. Prominent histopathological changes were observed in the liver, kidney, lung, and small intestine (Figure 6). The liver sections showed multiple foci of hepatocellular edema, and irregular vacuoles were observed in the cells. Histopathological changes in the small intestine consisted of necrosis of the intestinal mucosa, disappearance of intestinal glands, and destruction of tissue structure accompanied by massive hemorrhage. In addition, a large number of red blood cells were observed in the tubulointerstitial and alveolar tissues, and renal tubular protein casts could be observed. However, for the brain section, the vasodilatation and congestion of the blood vessel around meninges has been found, but no significant damage for brain neuron cells.

### Human Seroprevalence Study for EBIV

The average age of the 211 participants was 36.9 years (standard deviation = 22.4). The male/female ratio was 1.45:1. The majority of the study participants were farmers and 38.4% (81/211) were factory workers, followed by 22.3% (47/211) soldiers, 20.4% (43/211) children and students, and 18.9% (40/211) retirees. Detailed characteristics of the tested sera are presented in Table 1.

**TABLE 1:** Study participant characteristics and human serum samples positive for EBIV.

Group	Positive rate (%)		
	IgM 1:4 (IFA)	IgG 1:10 (IFA)	PRNT90
**Gender**			
Male	4.00(5/125)	9.6(14/125)	0.8(1/125)
Female	13.95(12/86)	13.95(12/86)	1.16(1/86)
**Age group (years)**			
0–20	9.52(4/52)	11.9(6/52)	0(0/52)
21–40	4.34(1/60)	9.52(5/60)	0(0/60)
41–60	9.52(6/63)	11.11(7/63)	3.17(2/63)
>60	16.67(6/36)	12.69(8/63)	0(0/63)
**Occupation**			
Child and student	9.3(4/43)	11.63(5/43)	0(0/43)
Farmer and factory worker	6.17(5/81)	17.28(14/81)	2.47(2/81)
Soldier	0(0/47)	4.26(2/47)	0(0/47)
Retiree	20(8/40)	12.5(5/40)	(0/40)
**With fever symptoms**			
No	0(0/47)	4.26(2/47)	0(0/47)
Yes	10.37(17/164)	14.63(24/164)	1.22(2/164)
Total	8.05(17/211)	12.3(26/211)^a^	0.95(2/211)^b^

*^*a*^The IgM-positive population was not the same as the IgG-positive population; only four cases were both IgM and IgG positive. ^*b*^These two PRNT-positive patients were positive for IgG but not for IgM.*

Of the 211 tested serum samples, 17 were identified as IgM positive (1:4), 26 as IgG positive (1:10), and 4 as both IgM and IgG positive for EBIV by IFA (Supplementary Figure 3). In the participant with fever, higher IgM (10.37% vs. 0) and IgG (14.63 vs. 4.26%) positive rates were observed. Female participants had a much higher IgM-positive rate than male participants (13.95 vs. 4.00%), but no obvious difference was observed for IgG (13.95 vs. 9.6%). Study participants belonging to the >60-year age group had the highest positive rate of both IgM (16.67%) and IgG (12.69%) among all age groups. Furthermore, based on the occupational groups, the proportion of positive samples for IgM and IgG was highest among retirees (20%), followed closely by farmers and factory workers (17.28%). In addition, the neutralizing antibody prevalence of EBIV was 0.95% (2/211) (Table 1). For the two neutralizing antibody-positive cases, one male participant aged 50 years had a 1:8 PRNT90 titer, and the second case was from a female participant aged 50 years with a 1:16 PRNT90 titer. Both of these two study participants were from the Fifth Division of Xinjiang Production and Construction Corps region.

## Discussion

The present study further characterized EBIV, which was previously isolated from *C. modestus* mosquitoes collected from the Ebinur Lake region. The EBIV segments S and M showed considerable genetic distance to currently known viruses, with 11 and 19% distance from the NP and G proteins of its closest relative, GERV. In contrast to dengue virus, Japanese encephalitis virus and other mosquito-borne flaviviruses, orthobunyaviruses are quite underestimated with regard to their potential prevalence and distribution in mainland China. To the best of our knowledge, Batai virus from the Bunyamwera serogroup and Tahyna virus from the California encephalitis serogroup are the only two orthobunyaviruses that have been documented in China (Lu et al., 2009; Liu et al., 2011, 2014a; Xia et al., 2018b). Hence, describing a newly identified *Orthobunyavirus* member is beneficial in understanding the diversity of orthobunyaviruses in China.

The genetic reassortment of viruses with triplicate segments in the order *Bunyavirales* plays an important role in driving the diversity and evolution of this virus group. For example, Nigari virus, which was characterized as a progeny from parental Batai virus and Bunyamwera virus, was implicated in hemorrhagic fever outbreaks in East Africa (Briese et al., 2006). In addition, natural M segment reassortment has been observed in Potosi and Main Drain viruses (Briese et al., 2007). In the present study, a potential reassortment signal of the M segment was observed through the different phylogenetic tree topologies of the M segment sequences compared to the S and L segment sequences. Moreover, a small M segment recombination event was observed between EBIV, Birao virus, and Northway virus, which are viruses identified from China, Africa, and North America, respectively. The long-distance spread of these viruses may be due to migratory birds. Because there are two migratory bird flyways (Central Asian flyway and West Asian–East African flyway) that pass or cover the region of Xinjiang (Olsen et al., 2006), the viruses could have been carried by birds from Africa or America to China, and then, the recombination event occurred in local invertebrate or vertebrate hosts. The other probable possibility is that Xinjiang spans over 1.6 million km^2^, with no systematic research and surveillance studies for mosquito-borne virus; hence, other orthobunyaviruses that are similar to the Northway or Birao virus might be circulating in Xinjiang and are currently not identified or underreported. The recombinant region is located at the beginning of Gc in the glycoprotein. The N-terminal domain of Gc of viruses in the Bunyamwera serogroup is not essential for the infection of and replication in cultured cells, but the N-terminal domain does play some role in the infection process (Shi et al., 2009). This recombination event may have enhanced or impaired the viral ability to infect natural hosts. However, due to the small quantity of available virus sequences used in the analysis and the low nucleotide identity between EBIV and other members in the Bunyamwera serogroup, much more work should be done to clearly provide evidence of the reassortment and recombination events in EBIV.

Genetic analysis of EBIV based on its S and M segments showed that it was closely related to GERV, a virus that was isolated almost 50 years ago from *Aedes circumluteolus* mosquitoes and rodents in South Africa and is associated with human infections (Monath et al., 1972; Venter, 2018). From our findings, *in vitro* tests indicate that the virus grew faster and caused more obvious CPEs in mammalian cell lines while causing a persistent infection in mosquito cells without obvious CPEs. These findings are in agreement with other previously reported studies on members of the Bunyamwera serogroup. The viral NSs protein could be one of the factors responsible for the different infection outcomes of orthobunyaviruses in mammalian and mosquito cell lines (Szemiel et al., 2012). The *in vivo* infection experiment showed that EBIV causes a lethal infection in adult mice through i.p. route. This finding corresponds with the reported results on GERV, but detailed information on the pathology changes of mice to GERV infection is not available (Monath et al., 1972). There are reports indicating that several members of orthobunyaviruses are neurovirulence in lab experimental animals, such as mice and hamsters. High viral titers could be detected in the brain tissue of sick mice and exhibit paralysis and convulsion signs (Wilson et al., 2015). In this study, the significant CNS symptoms such as paralysis, ataxia, or convulsion and the damage for brain neuron cells has not been detected in EBIV-infected Kunming mice. Since the EBIV presenting 10–30% genetic divergence with the other bunyamwera serogroup member, EBIV could have difference neurovirulence or neurotropism features in mice. Therefore, more in-depth studies should be conducted to fully comprehend the pathogenesis and immune response of EBIV in different animal models.

In human seroepidemiological surveys, a cross-reaction could occur in the IFA or PRNT test, and no other confirmatory data, such as positive RT-PCR results, viral isolation, or later convalescent sera test results, were available; therefore, no clinical conclusions for this human infection for EBIV can be drawn. However, these results provide us with some insights into EBIV infection in local residents, providing new ideas for clinicians and researchers to diagnose unknown fever cases in this region. We will follow up on these results to determine whether EBIV could cause human cases in this region with the following measures: (1) neutralization test for patients with acute and convalescent sera to see if the neutralizing antibody titer is increased by four or more times; (2) virus isolation in serum samples from patients in the acute phase; and (3) nucleic acid detection of patient specimens in the acute phase. Furthermore, the presence of EBIV nucleic acids and antibodies in local birds and vertebrates will be conducted to help us understand the natural cycle of EBIV in this region.

The Xinjiang region is demarked as the main gateway of China’s Silk Road economic corridor to Central Asia and Europe. As a result, this region is experiencing rapid growth in terms of infrastructure and human population development. Consequently, the deforestation rate has extensively increased in recent years. In turn, this scenario has augmented the risk for the emergence of zoonoses. Most importantly, the Ebinur Lake Nature Reserve is located at the China–Kazakhstan border, a region with rich biodiversity that provides an appropriate environment for the virus–mosquito–host cycle. Therefore, more surveillance and experimental transmission studies should be conducted in this region to determine the threat of this neglected EBIV to animal and human health.

## Data Availability Statement

Publicly available datasets were analyzed in this study. This data can be found here: KJ710424, KJ710423, KJ710425, MH484281, MH484280, MH484279, KU746869, KU746870, KU746871, MH370823, MH370822, MH370821, MH370826, MH370825, MH370824, NC_001927, NC_001926, NC_001925, KX100133, KX100134, KX100135, MH484296, MH484295, MH484294, M19420, M21951, KF234073, KF234074, KF234075, NC_038740, NC_038739, NC_038738, MH484305, MH484304, MH484303, KY910431, KY910430, KY910429, MH484308, MH484307, MH484306, KJ716848, KJ716849, KJ716850, MH484314, MH484313, MH484312, KX100121, KX100122, KX100123, MH484323, MH484322, MH484321, MH484332, MH484331, MH484330, MH484335, MH484334, MH484333, MH484344, MH484343, MH484342, KX817314, KX817313, KX817312, MG765471, MG765470, MG765469, KT288271, KT288270, KT288269, KX817335, KX817334, KX817333, NC_004110, NC_004109, NC_004108, KX817332, KX817331, KX817330, EU294510, EU262553, EU203678, KX817338, KX817337, KX817336, HM243139, HM243138, HM243137, KX891323, KX891322, KX891321, MG029274, MG029273, MG029272, NC_034498, NC_034505, NC_034497, MG029283, MG029282, MG029281, MG029292, MG029291, MG029290, NC_038725, NC_038723, NC_038724, NC_038726, NC_038727, NC_038728, NC_038737, NC_038735, NC_038736, KJ481927, KJ481928, KJ481929, KJ481921, KJ481922, KJ481923, NC_026282, NC_026283, NC_026281, NC_022597, NC_022596, NC_022595, MH017269, MH017281, MH017275, MH017270, MH017282, MH017276, MH017273, MH017283, MH017277, MH017271, MH017284, MH017278, NC_009896, NC_009895, NC_009894, JQ675601, JQ675602, JQ675603, MH484338, MH484337, MH484336, MF926352, MF926353, MF926354, NC_018462, NC_018466, NC_018461, NC_018464, NC_018467, NC_018463, NC_018460, NC_018459, NC_018465, NC_018477, NC_018478, NC_018476, NC_038712, NC_038713, and NC_038714.

## Ethics Statement

The studies involving human participants were reviewed and approved by the Ethical Committee of the Center for Disease Control and Prevention of Xinjiang Military Command Region Approval No. 2014006. Written informed consent to participate in this study was provided by the participants’ legal guardian/next of kin. The animal study was reviewed and approved by the Animal Care and Use Committee of the Center for Disease Control and Prevention of Xinjiang Military Command Region Approval No. 2014001.

## Author Contributions

ZY, HX, and GZ designed the experiments. HX, RL, and LZ performed the experiments. HX, LZ, and EA analyzed the data. ZY, XS, ZZ, and XH contributed the reagents, materials, and analysis tools. LZ, HX, EA, GZ, and ZY wrote the manuscript. BZ wrote and reviewed the manuscript.

## Conflict of Interest

RL is employed by Illumina, China. The remaining authors declare that the research was conducted in the absence of any commercial or financial relationships that could be construed as a potential conflict of interest.

## Footnotes

1 http://web.cbio.uct.ac.za/~brejnev/

2 https://www.megasoftware.net/

3 https://github.com/ddarriba/jmodeltest2

4 http://web.cbio.uct.ac.za/~darren/rdp.html

5 https://www.r-project.org/
