# Supplementary material for: Characterization of Ebinur Lake Virus and Its Human Seroprevalence at the China–Kazakhstan Border
Source: Front Microbiol. 2020 Jan 30;10:3111. doi: 10.3389/fmicb.2019.03111 (PMC7002386; doi:10.3389/fmicb.2019.03111)
Supplement: Supplementary file 1 [file Data_Sheet_1.pdf]

Supplementary Material

Supplementary Tables：

Supplementary Table 1. The background information for viruses used for phylogenetic analyses in this study

Genus	Serogroup	Viruses	Strain	Location	Date	Isolated source	Accession No. S	Accession No. M	Accession No. L
Orthobunyavirus	Bunyamwera	Ebinur lake orthobunyavirus	Cu20-XJ	China: Xinjiang	2013	Culex modestus	KJ710424	KJ710423	KJ710425
		Anadyr virus	LEIV 13395Mg	NA	1989	NA	MH484281	MH484280	MH484279
		Batai virus	ZJ2014	China: Zhejiang	2014	duck	KU746869	KU746870	KU746871
		Birao virus	DakArB 2198	Central African Republic: Banugui	1969	Anopheles pharoensis	MH370823	MH370822	MH370821
		Bozo virus	DakArB 7343	Central African Republic: Bozo	1975	Aedes opok	MH370826	MH370825	MH370824

	Bunyamwera virus	prototype	Uganda	1946	mosquito	NC_001927	NC_001926	NC_001925
	Cache Valley virus	6V633	USA	1955	Culiseta inornata	KX100133	KX100134	KX100135
	Fort Sherman virus	86MSP18	Panama	1985	human serum	MH484296	MH484295	MH484294
	Germiston virus	NA	Uganda	1968	rodents/ mosquito	M19420	M21951	NA
	Ilesha virus	R5964	NA	NA	NA	KF234073	KF234074	KF234075
	Kairi virus	BeAr8226	Brazil	1957	Ochlerotatus scapularis	NC_038740	NC_038739	NC_038738
	Lokern virus	A 10391	USA	NA	NA	MH484305	MH484304	MH484303
	Maguari virus	BeAr 7272	Brazil: Utinga forest	1957	mosquito	KY910431	KY910430	KY910429
	Main Drain virus	72V2567	USA: New Mexico	1972	Aedes vexans	MH484308	MH484307	MH484306

	Ngari virus	Adrar	Mauritania	2010	small ruminant	KJ716848	KJ716849	KJ716850
	Northway virus	243	USA: Alaska, Northway	1971	Aedes sp.	MH484314	MH484313	MH484312
	Playas virus	75V3066	Ecuador	1975	Aedes taeniorhynchus	KX100121	KX100122	KX100123
	Potosi virus	89-3380	USA: Potosi, Missouri	1989	Aedes albopictus	MH484323	MH484322	MH484321
	Shokwe virus	SAAr 4042	South Africa: Ndumu, Natal, Shokwe pan	1962	Aedes cumminsii	MH484332	MH484331	MH484330
	Tensaw virus	A9-171B	USA: Baldwin County, Alabama	1960	Anopheles crucians	MH484335	MH484334	MH484333
	Tlacotalpan virus	61D240	Mexico: Tlacotalpan, Veracruz	1961	Mansonia titillans	MH484344	MH484343	MH484342
California	California encephalitis virus	BFS 283	USA: California	1943	Aedes dorsalis	KX817314	KX817313	KX817312
	Keystone virus	AVA1709441	USA: Orange county, Texas	2017	Culex sp.	MG765471	MG765470	MG765469

	Inkoo virus	LEIV-15248Iv	Russia: Ivanovo district	1973	Aedes sp.	KT288271	KT288270	KT288269
	Serra do Navio virus	BeAr 103645	Brazil: Ampa	1966	Aedes fulvus	KX817335	KX817334	KX817333
	La Crosse virus	Human/78	NA		human	NC_004110	NC_004109	NC_004108
	San Angelo virus	20230	USA: Texas	1958	Anopheles pseudopunctipennis	KX817332	KX817331	KX817330
	Snowshoe hare virus	original	USA: Montana	1959	hare brain	EU294510	EU262553	EU203678
	South River virus	NJO-94F	USA: New Jerse	1960	Anopheles crucians	KX817338	KX817337	KX817336
	Tahyna virus	XJ0708	China: Xinjiang	2007	Aedes vexans	HM243139	HM243138	HM243137
	Trivittatus virus	AR42	USA: Denton County, Texas	2016	Aedes trivittatus	KX891323	KX891322	KX891321
Group C	Caraparu virus	BeAn3994	Brazil	1956	Sapajus apella	MG029274	MG029273	MG029272

	Madrid virus	BT4075	Panama	1956	human	NC_034498	NC_034505	NC_034497
	Marituba virus	BeAn15	Brazil	1954	Sapajus apella	MG029283	MG029282	MG029281
	Oriboca virus	BeAn17	Brazil	1954	Sapajus apella	MG029292	MG029291	MG029290
Guama	Bimiti virus	TRVL 8362	Russian: Trinidad and Tobago	1955	Culex spissipes	NC_038725	NC_038723	NC_038724
	Catu virus	BeH 151	Brazil	1955	human	NC_038726	NC_038727	NC_038728
	Guama virus	BeAn 277	Brazil	1955	Cebus apella	NC_038737	NC_038735	NC_038736
Mapputta	Buffalo Creek virus	DPP186	Australia	1982	Anopheles meraukensis	KJ481927	KJ481928	KJ481929
	Mapputta virus	MRM186	Australia	1960	Anopheles meraukensis	KJ481921	KJ481922	KJ481923

	Maprik virus	MK7532	Australia	1966	Verrallina funerea complex sp.	NC_026282	NC_026283	NC_026281
	Murrumbidgee virus	934	Australia	1997	Anopheles annulipes	NC_022597	NC_022596	NC_022595
Patois	Abras virus	75V1183	Ecuador	1974	Culex paracrybda	MH017269	MH017281	MH017275
	Babahoyo virus	75V2858	Ecuador	1975	Culex ocossa	MH017270	MH017282	MH017276
	Patois virus	63A49	Mexico	1963	Culex sp.	MH017273	MH017283	MH017277
	Shark river virus	64U80	Mexico	1964	sentinel hamster	MH017271	MH017284	MH017278
Simbu	Akabane virus	OBE-1	Japan:Okayama	1974	Bovine fetus	NC_009896	NC_009895	NC_009894
	Jatobal virus	BeAn 423380	Brazil	1984	Nasua	JQ675601	JQ675602	JQ675603

	Thimiri orthobunyavirus	VRC 66414	India: Thimiri, Walljah Tk., N. Arcot District	1963	Ardeola grayii	MH484338	MH484337	MH484336
	Oropouche virus	OROV/EC/Esmeraldas/087/2016	Ecuador	2016	human	MF926352	MF926353	MF926354
	Sathuperi virus	BH80/11-4	NA	2011	NA	NC_018462	NC_018466	NC_018461
	Shamonda virus	Ib An 5550	NA	NA	NA	NC_018464	NC_018467	NC_018463
	Aino virus	38K	NA	NA	NA	NC_018460	NC_018459	NC_018465
	Simbu virus	SA Ar 53	NA	NA	NA	NC_018477	NC_018478	NC_018476
Herbert herbevirus	Herbert virus	F23/CI/2004	Cote d'Ivoire	2004	Culex nebulosus	NC_038712	NC_038713	NC_038714

Supplementary Table 2. Putative reassortment/recombination event of the M segment among EBIV and members of Bunyamwera serogroup, predicted by RDP software v. 4.74

Recombinant *		Ebinur lake orthobunyavirus
Event		2
Initial Breakpoint Position		1584
Ending Breakpoint Position		1800
Genomic segment		M
Minor parent		Unknown (Birao virus)
Major parent		Northway virus
P-values determined by different algorithms	RDP	8.359E-3
	Bootscan	2.373E-4
	MaxChi	6.333E-3
	Chimaera	2.658E-2

* Possible misidentification of the recombinant was suggested by the software

** Statistical analysis was performed at an highest acceptable P-value of 0.05

Supplementary Figures:

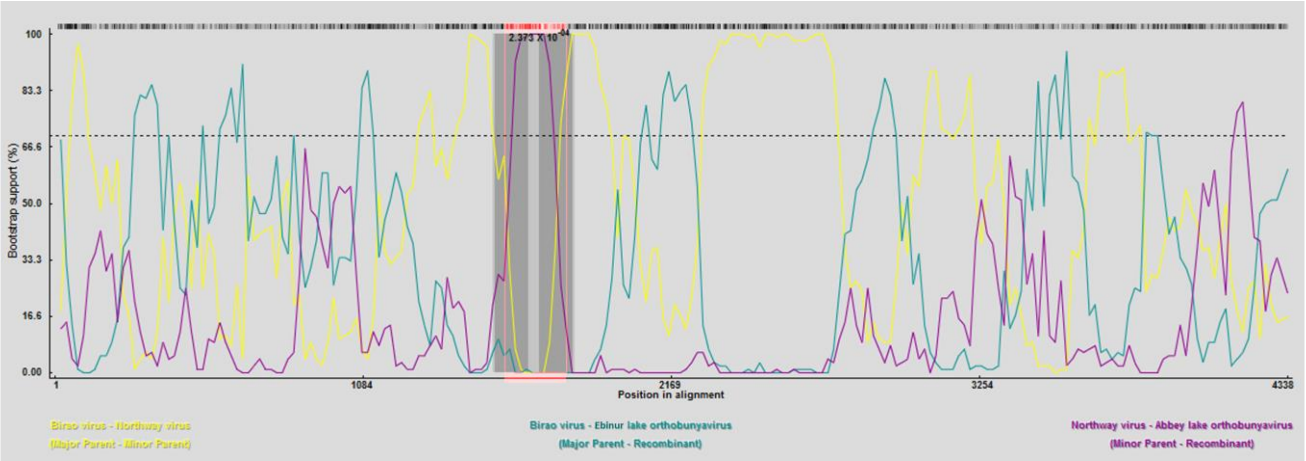

Supplementary Figure 1. Diagram for Putative recombination event of EBIV.

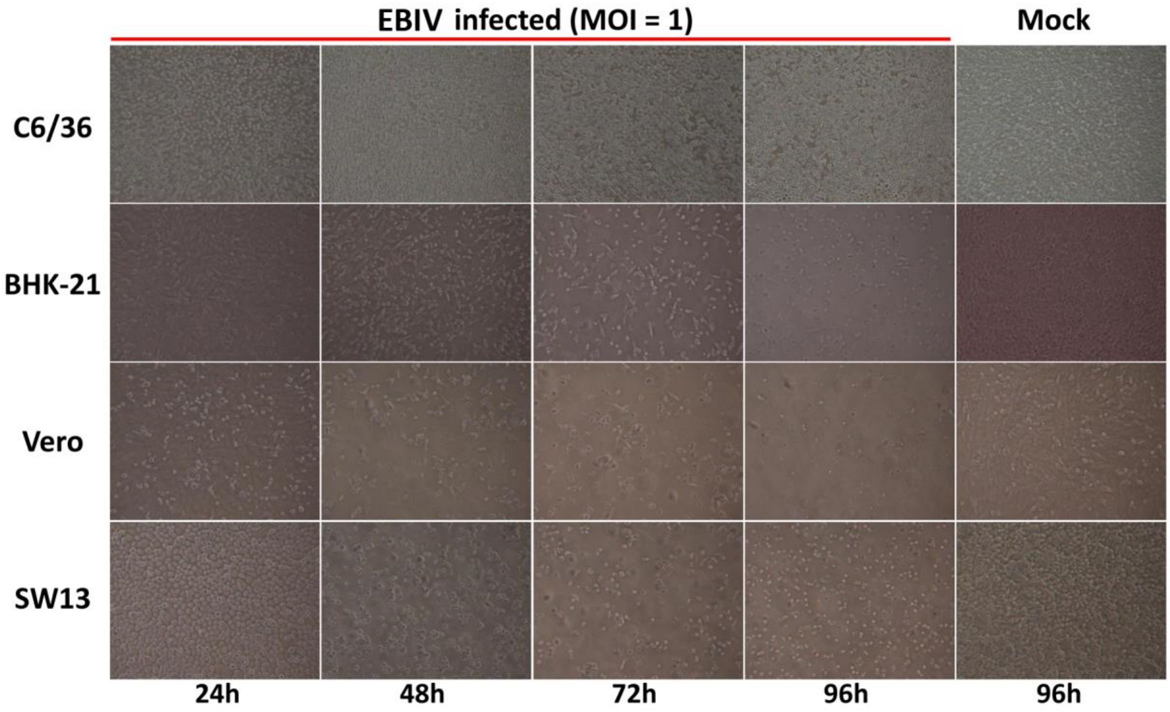

Supplementary Figure 2. Strong cytopathic effect observed in EBIV infected BHK-21, Vero, and SW13 cells

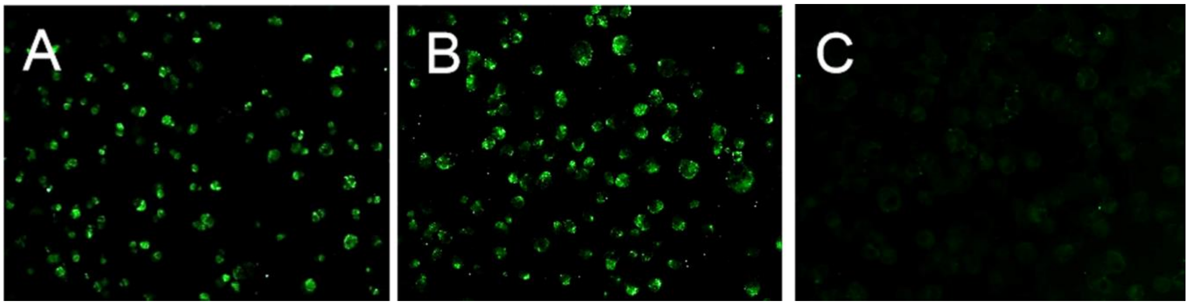

Supplementary Figure 3. Indirect Immunofluorescence assay for EBIV antibody in human serum. (A) IgM positive (B) IgG positive, and (C) negative control.
